# Flank Bulging and Sensory Disturbances After Open Renal Surgery Through Lumbotomy: Clinical Phenotypes and Procedure-Related Associations

**DOI:** 10.3390/medicina62101838

**Published:** 2026-09-23

**Authors:** Adam Majchrzak, Tomasz Ząbkowski, Kamil Obel, Marcin Talaga, Jolanta Łuniewska-Bury, Tomasz W. Kaminski, Tomasz Syryło

**Affiliations:** 1 Department of General, Functional and Oncological Urology, Military Institute of Medicine—National Research Institute, 128 Szaserów Street, 04-141 Warsaw, Poland; amajchrzak2@wim.mil.pl (A.M.);; 2 Warsaw Bar Association, 15/16 Żytnia Street, 01-014 Warsaw, Poland; 3 Faculty of Medicine, Collegium Medicum, The Mazovian University in Płock, 2 Pl. Dąbrowskiego Street, 09-402 Płock, Poland; 4 Hemostasis and Thrombosis Program, Versiti Blood Research Institute, Milwaukee, WI 53226, USA

**Keywords:** lumbotomy, open renal surgery, flank bulge, scar hypoesthesia

## Abstract

*Background and Objectives*: Flank bulging is a recognized consequence of open renal surgery through a lumbotomy, but its clinical relevance and relationship with postoperative sensory changes are not well defined. We assessed the frequency and clinical burden of postoperative flank bulging, its overlap with sensory disturbances, and procedure-related factors associated with its occurrence. *Materials and Methods*: We reviewed 407 patients who underwent open renal surgery through a subcostal lumbotomy between 2018 and 2023. Flank bulging was the primary outcome. We also recorded scar hypoesthesia, wider areas of sensory loss, and whether patients found the resulting deformity bothersome. Factors associated with bulging were explored using multivariable logistic regression, with a separate analysis comparing nephron-sparing surgery (NSS) and radical nephrectomy. *Results*: Flank bulging was found in 88 patients (21.6%). Scar hypoesthesia occurred in 14.0% and more extensive sensory loss in 7.1%. Sensory abnormalities showed a strongly nested clinical pattern: all patients with scar hypoesthesia had flank bulging, and all extended sensory deficits occurred among patients with both bulging and scar hypoesthesia. All patients with scar hypoesthesia also had bulging, while wider sensory loss was seen only when both were present. Among patients for whom this information was available, 52.9% described the bulging as bothersome. It was more common after NSS than after radical nephrectomy (27.0% vs. 16.3%). The difference persisted after adjustment for age and sex (aOR 2.05, 95% CI 1.17–3.59), corresponding to an adjusted absolute risk difference of 11.8 percentage points (95% CI 2.4–20.8). *Conclusions*: About one in five patients developed flank bulging after open renal surgery through a lumbotomy, and many of those affected found it bothersome. The accompanying sensory changes raise the possibility that nerve injury contributes to this complication, although our data cannot establish the underlying mechanism. The higher rate observed after NSS compared with radical nephrectomy also warrants further study. Prospective assessment with standardized imaging and more detailed operative data may help explain these findings and identify factors that could reduce the risk of postoperative bulging.

## 1. Introduction

Lumbotomy incisions provide direct access to the retroperitoneal space and continue to be regarded as a valuable operative option in urological surgery, particularly in complex scenarios or when minimally invasive techniques are not feasible [1]. At the same time, flank access requires dissection through the lateral abdominal wall and may expose the intercostal and subcostal nerve branches to injury, particularly during muscle division and retraction [2].

Clinically, such nerve injuries may manifest as sensory disturbances, chronic neuropathic pain, and the development of a so-called flank bulge, which is defined as asymmetry or a protrusion of the lateral abdominal wall. Recent studies have estimated that this complication occurs in approximately 25% of patients following a lumbar incision, and nearly half of the affected individual’s patient-reported functional limitation or cosmetic dissatisfaction [3,4].

The extent of this complication is influenced by several factors, including whether the approach is intercostal or subcostal, the degree of dissection and retraction, and patient-specific anatomical conditions. Prospective imaging studies in patients undergoing posterior retroperitoneoscopic adrenalectomy have demonstrated that subcostal nerve injury may occur following retroperitoneal access and can be evaluated using high-resolution ultrasound [5]. At the same time, both surgical and urological literature emphasize that the so-called “pseudohernia,” resulting from injury to the nerves of the lateral abdominal wall without fascial disruption, is not an uncommon finding after intercostal incisions. Similar complications have been documented in other surgical specialties, such as spinal procedures and thoracic surgery, supporting the concept of a shared pathophysiological mechanism driven by denervation [6,7].

Recently, there has been an increasing emphasis on standardizing the evaluation of abdominal wall complications. The proposed classifications, which incorporate computed tomography imaging together with patient-reported outcome measures (PROMs), make it possible to objectively assess both the severity of deformities and their functional impact. This framework facilitates the identification of patients who may benefit from targeted rehabilitation, neuromodulation strategies, or surgical abdominal wall reconstruction [3,4].

In addition, preventive strategies have been continuously refined. Anatomical and clinical research has highlighted the concept of the so-called “safe line,” in which the incision is placed above the line connecting the apex of the 12th rib with the umbilicus, thereby reducing the risk of injury to the T11–T12 intercostal and subcostal nerves. Additional measures, such as limiting the duration and force of retraction, preserving muscular attachments, and exercising caution during partial resection of the 12th rib, further lowered the likelihood of abdominal wall denervation [8,9].

Minimally invasive renal surgery may reduce access-related abdominal wall morbidity. In a direct comparison of open and laparoscopic partial nephrectomy, laparoscopic surgery was associated with significantly lower rates of postoperative flank bulging, paresthesia, and numbness, as well as less loss of flank muscle volume [10]. Nevertheless, an open approach remains necessary in selected patients in whom minimally invasive surgery may not be feasible or appropriate. Contemporary reviews and analyses of open nephrectomy series emphasize that a flank bulge and sensory deficits remain clinically relevant sequelae, with the potential to negatively affect functional capacity and patient-reported satisfaction [11,12]. Persistent neuralgia, particularly involving the genitofemoral nerve, may arise when the nerve is entrapped by a surgical clip or stapler. In such cases, targeted surgical exploration with subsequent neurectomy has been shown to provide effective symptom relief [2,13].

We hypothesized that flank bulging and postoperative sensory disturbances may represent overlapping manifestations of abdominal wall morbidity after lumbotomy. We also explored whether their frequency differed between renal procedures, as different operations may involve differences in surgical exposure, dissection, and retraction despite the use of the same general lumbotomy approach. Procedure type was therefore considered a potential marker of differences in operative exposure rather than a presumed direct cause of these complications.

Given the inconsistencies in the literature, including variations in surgical techniques, outcome definitions, and follow-up periods, there is a need for larger clinical studies integrating abdominal wall findings with patient-reported symptoms and sensory outcomes.

The present study provides a structured analysis of a large consecutive cohort, allowing the frequency and clinical pattern of abdominal wall complications after lumbotomy to be examined in greater detail. By evaluating flank bulging, scar hypoesthesia, and extended sensory disturbances within the same cohort, we aimed to determine how often these findings occur together and whether their frequency differs between renal procedures. The observed overlap may be compatible with a shared underlying mechanism, including possible denervation, but the retrospective clinical data do not allow the underlying mechanism to be established.

This approach addresses important limitations of previous heterogeneous reports and contributes to improving the consistency of outcome assessment in this field.

## 2. Materials and Methods

This retrospective, single-center study included 407 consecutive patients who underwent open renal surgery via a lumbotomy approach between 2018 and 2023. Depending on the type of procedure performed, patients were divided into four groups: nephron-sparing surgery (NSS), radical nephrectomy, heminephrectomy, and nephroureterectomy.

Of the 407 patients included in the analysis, 189 underwent NSS and 141 underwent radical nephrectomy. Heminephrectomy was performed in 42 patients, while the remaining 35 underwent nephroureterectomy. Patients were excluded if they had previously undergone abdominal wall surgery, had a known neuromuscular disorder, or if their medical records were incomplete to an extent that did not allow reliable assessment of the postoperative outcomes analyzed in this study. Because the study was based on a pre-existing retrospective clinical database, the original screening log was not available; therefore, the exact number of patients initially assessed, and the number excluded according to each individual exclusion criterion could not be reliably reconstructed.

Clinical and perioperative data were obtained retrospectively from medical records and postoperative follow-up documentation. Data extraction was performed according to a predefined case report form. Whenever available, postoperative findings were cross-validated using outpatient follow-up records to reduce information bias. All included patients had documented postoperative follow-up of at least 6 months. Exact patient-level follow-up duration beyond this minimum was not consistently available in the analytical dataset and could not be reliably reconstructed.

The study was conducted in accordance with the Declaration of Helsinki and was approved by the Bioethics Committee of the Military Institute of Medicine–National Research Institute (approval No. 56/25; 15 October 2025). Because of the retrospective design and use of anonymized clinical data, the requirement for individual informed consent was waived in accordance with the decision of the institutional ethics committee.

### 2.1. Surgical Approach and Perioperative Variables

All procedures were performed through an open subcostal lumbotomy below the 12th rib, without rib resection. Self-retaining retractors were routinely used to provide adequate exposure of the kidney. Retraction time varied according to the complexity of the procedure and generally ranged from 1.5 to 3 h.

The variables collected for the analysis included age, sex, type of surgery, hematocrit (HCT), and serum creatinine. HCT and creatinine levels were assessed before and after surgery, allowing us to examine perioperative changes and explore whether baseline laboratory values were related to the development of postoperative abdominal wall complications.

Because of the retrospective nature of the study, some technical details of the procedures could not be reliably obtained from the operative records. These included the exact length and location of the incision in relation to the proposed anatomical “safe line,” the force applied during retraction, the extent of muscle division, and specific techniques used to preserve the nerves. As these data were not recorded consistently, they were not included in the regression analyses.

### 2.2. Assessment of Postoperative Abdominal Wall Outcomes

The primary outcome of the study was postoperative flank bulging, defined as a visible or palpable asymmetry or protrusion of the lateral abdominal wall noted during clinical follow-up. Sensory assessment was performed systematically in all patients during postoperative follow-up, regardless of the presence or absence of flank bulging, using the same standardized clinical examination protocol; the absence of a documented sensory abnormality therefore represented a negative finding rather than a missing sensory assessment.

Sensory assessment was performed systematically in all patients during postoperative follow-up, regardless of the presence or absence of flank bulging, using the same standardized clinical examination protocol. Sensation was assessed in the surgical scar area and the adjacent ipsilateral abdominal wall. Sensation was assessed by light-touch testing and compared with the corresponding area on the contralateral side. Scar hypoesthesia was defined as reduced sensation predominantly confined to the scar area, whereas an extended sensory deficit was defined as sensory loss extending beyond the scar to the surrounding ipsilateral abdominal wall. The absence of a documented sensory disturbance represented a negative finding on the standardized examination rather than a missing sensory assessment. For the present retrospective analysis, the results of these standardized sensory examinations were extracted from the patients’ follow-up records.

For patients who developed flank bulging, we additionally assessed its clinical impact. Bulging was considered bothersome when patients reported cosmetic discomfort or interference with everyday activities. No validated patient-reported outcome instrument was used for this assessment. This analysis was limited to patients with documented flank bulging for whom patient-reported information was available.

Additional imaging was available only in selected patients. In those who underwent ultrasonography, no true incisional hernia was identified. Computed tomography available for one patient showed thinning of the lateral abdominal wall muscles without fascial disruption. Because imaging was not performed systematically, flank bulging was treated as a clinical outcome, and individual cases could not be definitively classified as denervation-related pseudohernia or structural abdominal wall defects.

### 2.3. Statistical Analysis

Statistical analyses were performed using the complete original study dataset comprising 407 patients. Continuous variables were evaluated for distributional characteristics and are presented as median and interquartile range [25th–75th percentile] because several variables showed non-normal distributions. Categorical variables are presented as counts and percentages.

Differences in continuous variables among the four surgical groups were assessed using the Kruskal-Walli’s test. Differences in categorical baseline characteristics were evaluated using the chi-square test, as appropriate.

The frequency of postoperative abdominal wall outcomes was calculated as the number of patients with the documented outcome divided by the number of evaluable patients and is presented as a percentage with a 95% confidence interval (CI). Confidence intervals for binomial proportions were calculated using the Wilson method. Global differences in postoperative outcome frequencies across the four surgical groups were evaluated using the Fisher-Freeman-Halton exact test. When procedure-specific pairwise comparisons were performed for flank bulging, two-sided Fisher’s exact tests were used, followed by Holm correction for multiple comparisons.

The primary inferential analysis evaluated factors associated with flank bulging. Because flank bulging was a binary outcome, multivariable logistic regression was used. Age, sex, and surgical procedure were selected a priori as clinically relevant covariates and entered simultaneously into the primary model. Age was modeled per 10-year increase. Radical nephrectomy was used as the reference category for surgical procedure. Procedure-specific contrasts with radical nephrectomy were considered exploratory, as no individual between-procedure contrast was specified a priori. Results are presented as odds ratios (ORs) with corresponding 95% CIs and two-sided *p* values. The overall contribution of surgical procedure to the multivariable model was additionally assessed using a likelihood-ratio test comparing models with and without the three-level procedure indicator terms.

To assess the robustness of the primary model, a sensitivity model additionally included preoperative hematocrit and serum creatinine. Hematocrit was modeled per 5-percentage-point increase, whereas serum creatinine was modeled per 1 mg/dL increase. Postoperative hematocrit and creatinine were not included as predictors in these models because they occurred after the operative exposure and therefore were not considered appropriate baseline covariates for the assessment of factors associated with postoperative flank bulging.

A separate sensitivity analysis was restricted to the two largest surgical groups, NSS and radical nephrectomy. In this analysis, crude event rates, odds ratios, relative risks, and absolute risk differences were calculated. Logistic regression adjusted for age and sex was subsequently performed, followed by a second model additionally adjusted for preoperative hematocrit and serum creatinine.

Age- and sex-adjusted probabilities of flank bulging for NSS and radical nephrectomy were estimated by marginal standardization of the fitted logistic regression model over the observed covariate distribution. Adjusted absolute risk differences and adjusted relative risks were derived from these standardized probabilities. Confidence intervals for marginal estimates were obtained using 10,000 non-parametric bootstrap resamples.

Because the presence of bothersome bulging was clinically meaningful only among patients who developed flank bulging, this outcome was analyzed exclusively within the subgroup of patients with flank bulging and available symptom data.

Preoperative and postoperative hematocrit and serum creatinine concentrations were compared within each surgical group using the paired Wilcoxon signed-rank test. These analyses were considered descriptive perioperative analyses rather than primary predictors of postoperative abdominal wall morbidity.

All tests were two-sided. *p* value < 0.05 was considered statistically significant. Where correction for multiple pairwise comparisons were applied, both unadjusted and Holm-adjusted *p* values are reported. Given the retrospective observational design, all regression results were interpreted as associations rather than evidence of causality.

## 3. Results

A total of 407 patients undergoing open renal surgery via lumbotomy were included in the analysis. The cohort comprised 189 patients undergoing nephron-sparing surgery (NSS), 141 undergoing radical nephrectomy, 42 undergoing heminephrectomy, and 35 undergoing nephroureterectomy (Table 1).

Overall, 244 patients (60.0%) were male, and the median age was 64.0 years [interquartile range (IQR), 55.0–70.0]. The distribution of sex differed significantly among surgical groups (*p* = 0.001), with the highest proportion of male patients in the NSS group (69.3%) and the lowest in the heminephrectomy group (40.5%). Age distributions differed significantly across the four surgical groups overall (Kruskal–Wallis *p* < 0.0001). Descriptively, the heminephrectomy group had the lowest median age [50.0 years; IQR, 42.0–60.2], whereas the nephroureterectomy group had the highest median age [68.0 years; IQR, 62.5–72.0].

Preoperative and postoperative hematocrit and serum creatinine values also differed significantly among surgical groups (Table 1). Median preoperative hematocrit was 39.0% [IQR, 36.0–42.0] in the overall cohort and ranged from 35.0% [32.5–39.0] in the nephroureterectomy group to 40.0% [37.0–43.0] in the NSS group (*p* < 0.0001). Median preoperative creatinine was 1.00 mg/dL [0.90–1.20] overall and differed among groups (*p* = 0.001). Postoperative hematocrit and creatinine also showed significant between-group differences (both *p* < 0.0001).

### 3.1. Frequency of Postoperative Abdominal Wall Complications

Flank bulging was the most frequent postoperative abdominal wall complication and was identified in 88 of 407 patients (21.6%; 95% CI, 17.9–25.9%) (Table 2). Among patients with flank bulging and available symptom data, 46 of 87 (52.9%; 95% CI, 42.5–63.0%) considered the deformity bothersome.

Scar hypoesthesia was documented in 57 of 407 patients (14.0%; 95% CI, 11.0–17.7%), whereas an extended sensory deficit involving the scar and ipsilateral abdominal wall was present in 29 patients (7.1%; 95% CI, 5.0–10.0%). Thus, flank bulging affected approximately one in five patients, and more than half of evaluable affected patients reported that the bulging was bothersome.

The distribution of these complications according to surgical procedure is shown in Table 2 and Figure 1. Flank bulging occurred most frequently after NSS, affecting 51 of 189 patients (27.0%; 95% CI, 21.2–33.7%). Corresponding rates were 16.3% (23/141; 95% CI, 11.1–23.3%) after radical nephrectomy, 19.0% (8/42; 95% CI, 10.0–33.3%) after heminephrectomy, and 17.1% (6/35; 95% CI, 8.1–32.7%) after nephroureterectomy. The global comparison across all four procedures did not reach statistical significance (exact *p* = 0.115).

Among evaluable patients who developed flank bulging, the proportion reporting clinically bothersome symptoms was 48.0% (24/50) after NSS, 65.2% (15/23) after radical nephrectomy, 62.5% (5/8) after heminephrectomy, and 33.3% (2/6) after nephroureterectomy. These differences were not statistically significant (global exact *p* = 0.378).

Scar hypoesthesia occurred in 16.4% of patients after NSS, 12.8% after radical nephrectomy, 11.9% after heminephrectomy, and 8.6% after nephroureterectomy, with no significant difference among procedures (global exact *p* = 0.629). Similarly, extended sensory deficits were observed in 7.9%, 6.4%, 7.1%, and 5.7% of patients, respectively, without evidence of a procedure-specific difference (global exact *p* = 0.968) (Table 2, Figure 1).

### 3.2. Clinical Overlap of Abdominal Wall and Sensory Phenotypes

The postoperative manifestations showed a marked hierarchical overlap (Figure 2). Of the 407 patients, 319 (78.4%) had neither flank bulging nor a sensory deficit. Thirty-one patients (7.6%) had flank bulging without documented sensory involvement, 28 (6.9%) had flank bulging accompanied by scar hypoesthesia, and 29 (7.1%) demonstrated the most extensive phenotype, comprising flank bulging, scar hypoesthesia, and an extended sensory deficit.

Accordingly, all 57 patients with scar hypoesthesia were contained within the group of 88 patients with flank bulging, representing 64.8% of patients with bulging. All 29 patients with extended sensory deficits were contained within the subgroup with scar hypoesthesia, corresponding to 50.9% of patients with scar hypoesthesia (Figure 2). Thus, sensory abnormalities were strongly nested within the flank-bulging phenotype, with extended sensory involvement confined to patients who also had localized scar hypoesthesia.

### 3.3. Multivariable Analysis of Factors Associated with Flank Bulging

To determine whether the observed differences in flank-bulging frequency persisted after accounting for demographic differences among surgical groups, multivariable logistic regression was performed with age, sex, and surgical procedure included a priori (Table 3). Radical nephrectomy served as the reference procedure.

The surgical procedure factor did not reach statistical significance in the global likelihood-ratio test (*p* = 0.065). In an exploratory procedure-specific comparison, NSS was associated with higher adjusted odds of flank bulging than radical nephrectomy (aOR, 1.99; 95% CI, 1.14–3.48; *p* = 0.016). In contrast, neither heminephrectomy (aOR, 1.00; 95% CI, 0.40–2.54; *p* = 0.995) nor nephroureterectomy (aOR, 1.16; 95% CI, 0.43–3.16; *p* = 0.765) was significantly associated with flank bulging relative to radical nephrectomy.

Age was not independently associated with flank bulging (OR per 10-year increase, 0.87; 95% CI, 0.70–1.07; *p* = 0.191). Similarly, male sex was not significantly associated with this outcome (OR, 0.86; 95% CI, 0.52–1.42; *p* = 0.556). The association between NSS and flank bulging remained stable in a sensitivity model additionally adjusted for preoperative hematocrit and serum creatinine (Table 3). In this model, NSS remained associated with higher odds of bulging compared with radical nephrectomy (aOR, 1.97; 95% CI, 1.12–3.46; *p* = 0.018). Neither preoperative hematocrit (OR per 5-percentage-point increase, 0.99; 95% CI, 0.75–1.30; *p* = 0.926) nor preoperative creatinine (OR per 1 mg/dL increase, 0.89; 95% CI, 0.53–1.52; *p* = 0.683) was associated with flank bulging.

### 3.4. Pairwise Comparisons of Flank Bulging Between Surgical Procedures

Because NSS showed the highest observed frequency of flank bulging, procedure-specific comparisons were additionally performed with radical nephrectomy as the reference group (Table 4).

In the unadjusted comparison, NSS was associated with higher odds of flank bulging than radical nephrectomy (OR, 1.90; 95% CI, 1.09–3.29; Fisher’s exact *p* = 0.0235). However, after Holm correction for the three procedure-specific comparisons, the association did not retain conventional statistical significance (Holm-adjusted *p* = 0.071).

No significant unadjusted differences were identified for heminephrectomy versus radical nephrectomy (OR, 1.21; 95% CI, 0.50–2.94; Fisher’s exact *p* = 0.646; Holm-adjusted *p* = 1.000) or nephroureterectomy versus radical nephrectomy (OR, 1.06; 95% CI, 0.40–2.85; Fisher’s exact *p* = 1.000; Holm-adjusted *p* = 1.000.

### 3.5. Perioperative Changes in Hematocrit and Serum Creatinine

Perioperative changes in hematocrit and creatinine were subsequently assessed within each surgical group (Table 5). Hematocrit decreased significantly following surgery in all four groups. In the NSS group, median hematocrit decreased from 40.0% [IQR, 37.0–43.0] to 35.0% [32.0–38.0] (*p* < 0.0001). Following radical nephrectomy, hematocrit decreased from 38.0% [36.0–41.0] to 35.0% [31.0–38.0] (*p* < 0.0001).

A smaller but statistically significant decrease was observed after heminephrectomy, from 39.0% [37.0–41.0] to 37.0% [35.0–39.75] (*p* < 0.0001). Following nephroureterectomy, median hematocrit decreased from 35.0% [32.5–39.0] to 31.0% [29.5–34.5] (*p* < 0.0001). Across the entire cohort, median hematocrit decreased from 39.0% [36.0–42.0] to 35.0% [31.0–38.0] (*p* < 0.0001).

Changes in serum creatinine differed according to surgical procedure. Following NSS, median creatinine increased modestly from 1.00 mg/dL [0.90–1.10] to 1.10 mg/dL [0.90–1.20] (*p* < 0.0001). A more pronounced increase was observed following radical nephrectomy, from 1.10 mg/dL [0.90–1.30] to 1.60 mg/dL [1.30–1.80] (*p* < 0.0001), and after nephroureterectomy, from 1.00 mg/dL [0.90–1.25] to 1.60 mg/dL [1.30–1.75] (*p* < 0.0001). In contrast, creatinine remained unchanged following heminephrectomy [1.00 mg/dL (0.90–1.18) before surgery vs. 1.00 mg/dL (0.90–1.10) after surgery; *p* = 0.588]. Across the entire cohort, median serum creatinine increased from 1.00 mg/dL [0.90–1.20] to 1.20 mg/dL [1.00–1.60] (*p* < 0.0001).

### 3.6. Sensitivity Analysis Restricted to NSS and Radical Nephrectomy

To further assess the robustness of the procedure-specific association, an additional sensitivity analysis was restricted to the two largest surgical groups: NSS and radical nephrectomy (n = 330). Flank bulging occurred in 51 of 189 patients undergoing NSS (27.0%) compared with 23 of 141 patients undergoing radical nephrectomy (16.3%). This corresponded to an unadjusted OR of 1.90 (95% CI, 1.09–3.29; *p* = 0.0235), a relative risk of 1.65 (95% CI, 1.06–2.53), and an absolute risk difference of 10.7 percentage points (95% CI, 1.6–19.2).

After adjustment for age and sex, NSS remained associated with approximately twice the odds of flank bulging compared with radical nephrectomy (aOR, 2.05; 95% CI, 1.17–3.59; *p* = 0.0127). Further adjustment for preoperative hematocrit and serum creatinine produced a nearly identical effect estimate (aOR, 2.02; 95% CI, 1.15–3.56; *p* = 0.0151).

Marginal standardization of the age- and sex-adjusted model estimated the probability of flank bulging at 27.6% following NSS compared with 15.8% following radical nephrectomy. This corresponded to an adjusted absolute risk difference of 11.8 percentage points (95% CI, 2.4–20.8) and an adjusted relative risk of 1.75 (95% CI, 1.12–2.91).

The direction and magnitude of the association between NSS and postoperative flank bulging were consistent across the primary and sensitivity analyses.

## 4. Discussion

Three findings emerge from this analysis. First, postoperative flank bulging was common after open renal surgery through lumbotomy, affecting approximately one in five patients, and more than half of evaluable affected patients considered the deformity bothersome. Second, sensory abnormalities showed a striking hierarchical overlap with flank bulging, with all cases of scar hypoesthesia occurring among patients with bulging and all extended sensory deficits occurring within this combined phenotype. Third, NSS was consistently associated with a higher occurrence of flank bulging than radical nephrectomy across adjusted and sensitivity analyses. Although the global comparison across all four procedures was not statistically significant and the unadjusted NSS-versus-radical-nephrectomy comparison did not remain significant after Holm correction, the exploratory adjusted analyses consistently yielded higher odds of flank bulging in the NSS group. This procedure-specific finding should therefore be interpreted cautiously and requires confirmation in future studies. Together, these findings characterize flank bulging as a clinically relevant component of postoperative abdominal wall morbidity rather than an exclusively cosmetic sequela of open renal surgery. The frequency of bulging observed in our cohort falls within the broad range reported after open retroperitoneal surgery, where estimates vary depending on the surgical approach, definition of the complication, and duration of follow-up [4,5,14,15,16,17].

The frequency of bulging also differed between the two most common procedures. It was observed in 27.0% of patients undergoing nephron-sparing surgery (NSS), compared with 16.3% after radical nephrectomy. Although no significant overall difference was found when all four procedures were considered together, the adjusted analysis showed nearly twice the odds of bulging after NSS compared with radical nephrectomy. This estimate changed very little after hematocrit and creatinine were added to the model. A separate analysis limited to NSS and radical nephrectomy led to a similar result, with an adjusted absolute difference in risk of 11.8 percentage points. Expressed clinically, the standardized estimates corresponded to approximately 12 additional cases of flank bulging per 100 patients undergoing NSS compared with radical nephrectomy.

It is difficult to determine from our data why this difference occurred. The same general subcostal approach below the 12th rib was used throughout the cohort, and none of the procedures involved rib resection. However, potentially relevant technical details, such as incision length, the extent and duration of retraction, muscle division, or nerve-preserving maneuvers, were not consistently documented. We therefore cannot determine whether the higher frequency observed in the NSS group was attributable to the renal procedure itself or to unmeasured differences in surgical exposure and technique. Incision characteristics, duration and extent of retraction, muscle division, nerve-preserving maneuvers, and other technical factors may have differed between procedures and could have contributed to the observed association. Accordingly, the comparison between NSS and radical nephrectomy should be interpreted as an association between procedure groups rather than as evidence of a procedure-specific causal effect.

The close relationship between bulging and sensory changes is also noteworthy. Every patient with scar hypoesthesia had flank bulging, and the broader sensory deficits occurred only in patients who also had bulging and scar hypoesthesia. This pattern may point towards a common neurological mechanism. Injury to the intercostal or subcostal nerve branches can result in denervation, followed by weakening and thinning of the lateral abdominal wall [9,18]. Comparable postoperative pseudohernias have been reported after other procedures involving lateral access, including thoracic and spinal surgery [7,19].

Our findings, however, do not allow all cases of flank bulging to be classified as denervation-related pseudohernias. Diagnosis was primarily clinical, and imaging was not routinely performed. No true fascial defect was found on ultrasonography in the selected patients who underwent additional assessment, while CT in one patient showed thinning of the abdominal wall musculature without fascial disruption. These observations support a possible denervation-related origin in at least some cases, but they cannot be extended to the entire cohort. A more reliable distinction between pseudohernia and incisional hernia would require systematic imaging.

The impact on patients is equally relevant. Of those who developed flank bulging and had available symptom data, 52.9% considered it bothersome. The problem should therefore not be viewed simply as a postoperative cosmetic change. For some patients, it may interfere with everyday functioning or become a source of persistent discomfort. Future studies would benefit from combining clinical and imaging findings with patient-reported outcomes to better capture this aspect of postoperative morbidity.

We did not find an independent association between flank bulging and either age or sex. Similarly, adding preoperative hematocrit and creatinine to the adjusted model had little effect on the association between surgical procedure and bulging. These baseline characteristics therefore do not appear to explain the higher frequency observed after NSS. Other factors that could not be captured retrospectively, particularly those related to surgical exposure and technique, may be more relevant.

Several limitations need to be considered. The retrospective, single-center design limits both causal interpretation and generalizability. In addition, the absence of the original screening log prevented reconstruction of the total number of patients initially assessed and the number excluded according to each eligibility criterion. Exact individual follow-up duration beyond the minimum 6-month postoperative assessment was not available in the analytical dataset, precluding formal adjustment for follow-up time. Variation in the duration of subsequent observation may therefore have influenced the likelihood of detecting or documenting flank bulging and may also have affected assessment of its evolution or recovery. Imaging was not performed systematically, making it impossible to reliably distinguish denervation-related bulging from a structural fascial defect in every patient. Operative records also lacked consistent information on several potentially important technical factors, including incision length, retraction, muscle division, and nerve preservation. Residual confounding also remains possible.

Nevertheless, the study provides data from a relatively large cohort of 407 patients managed with a consistent open lumbotomy approach. The higher frequency of flank bulging after NSS compared with radical nephrectomy was seen across the primary and sensitivity analyses, making this observation particularly relevant for further investigation. These findings are also relevant in the broader context of the continuing evolution of urological surgery toward minimally invasive and robotic approaches, which increasingly aim to reduce the morbidity associated with conventional open surgical access [20]. Prospective studies using standardized imaging, patient-reported measures, and more detailed operative data could help determine why these complications develop and, more importantly, whether any of the contributing factors can be modified.

## 5. Conclusions

Flank bulging was a common and clinically relevant complication after open renal surgery through lumbotomy, affecting approximately one in five patients and being considered bothersome by more than half of evaluable affected individuals. The marked overlap between bulging and sensory disturbances identifies a coherent postoperative phenotype potentially consistent with abdominal wall denervation, although the underlying mechanism requires objective confirmation. NSS was consistently associated with a higher occurrence of flank bulging than radical nephrectomy, supporting prospective investigation of procedure-specific and modifiable technical factors.

## Figures and Tables

**Figure 1:**
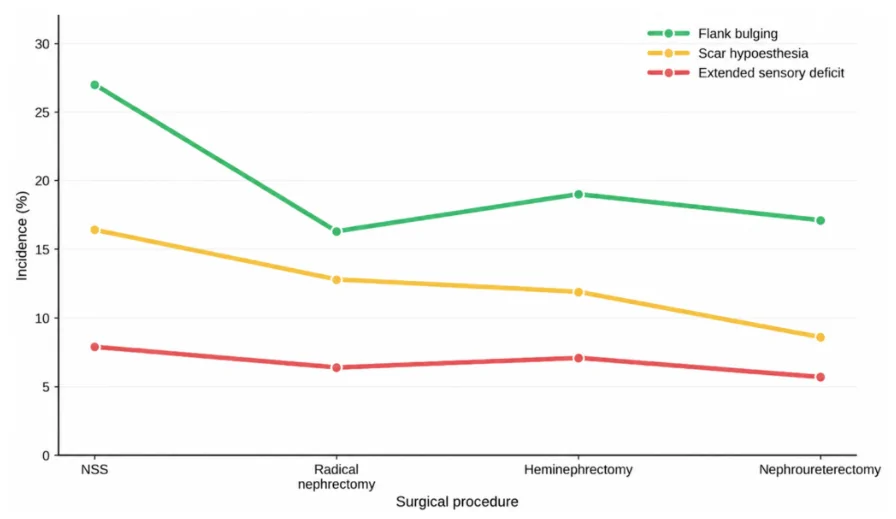
Frequency of postoperative abdominal wall outcomes by procedure.

**Figure 2:**
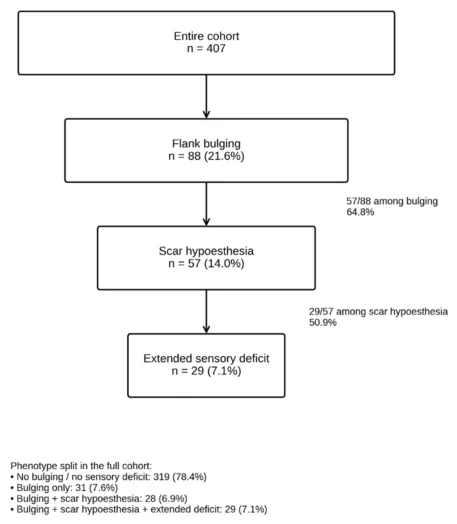
Nested phenotype hierarchy of postoperative abdominal wall complications.

**Table 1:** Cohort characteristics by surgical procedure.

Characteristic	Overall	NSS	Radical Nephrectomy	Heminephrectomy	Nephroureterectomy	Global *p* Value
N	407	189	141	42	35	
Male sex, n (%)	244 (60.0%)	131 (69.3%)	76 (53.9%)	17 (40.5%)	20 (57.1%)	0.001
Age, years, median [IQR]	64.0 [55.0–70.0]	65.0 [57.0–70.0]	64.0 [53.0–71.0]	50.0 [42.0–60.2]	68.0 [62.5–72.0]	<0.0001
HCT pre, %, median [IQR]	39.0 [36.0–42.0]	40.0 [37.0–43.0]	38.0 [36.0–41.0]	39.0 [37.0–41.0]	35.0 [32.5–39.0]	<0.0001
HCT post, %, median [IQR]	35.0 [31.0–38.0]	35.0 [32.0–38.0]	35.0 [31.0–38.0]	37.0 [35.0–39.8]	31.0 [29.5–34.5]	<0.0001
Creatinine pre, mg/dL, median [IQR]	1.00 [0.90–1.20]	1.00 [0.90–1.10]	1.10 [0.90–1.30]	1.00 [0.90–1.18]	1.00 [0.90–1.25]	0.001
Creatinine post, mg/dL, median [IQR]	1.20 [1.00–1.60]	1.10 [0.90–1.20]	1.60 [1.30–1.80]	1.00 [0.90–1.10]	1.60 [1.30–1.75]	<0.0001

**Table 2:** Postoperative abdominal wall outcomes.

Outcome	Group	Events	N	Rate (%)	95% CI Low	95% CI High	Global Exact *p*
Flank bulging	Overall	88	407	21.62	17.90	25.88	0.115
Flank bulging	NSS	51	189	26.98	21.16	33.72	
Flank bulging	Radical nephrectomy	23	141	16.31	11.12	23.29	
Flank bulging	Heminephrectomy	8	42	19.05	9.98	33.30	
Flank bulging	Nephroureterectomy	6	35	17.14	8.10	32.68	
Bothersome among bulging (evaluable)	Overall	46	87	52.87	42.49	63.02	0.377
Bothersome among bulging (evaluable)	NSS	24	50	48.00	34.80	61.49	
Bothersome among bulging (evaluable)	Radical nephrectomy	15	23	65.22	44.89	81.19	
Bothersome among bulging (evaluable)	Heminephrectomy	5	8	62.50	30.57	86.32	
Bothersome among bulging (evaluable)	Nephroureterectomy	2	6	33.33	9.68	70.00	
Scar hypoesthesia	Overall	57	407	14.00	10.97	17.71	0.628
Scar hypoesthesia	NSS	31	189	16.40	11.80	22.34	
Scar hypoesthesia	Radical nephrectomy	18	141	12.77	8.23	19.28	
Scar hypoesthesia	Heminephrectomy	5	42	11.90	5.19	25.00	
Scar hypoesthesia	Nephroureterectomy	3	35	8.57	2.96	22.38	
Extended sensory deficit	Overall	29	407	7.13	5.01	10.05	0.967
Extended sensory deficit	NSS	15	189	7.94	4.87	12.68	
Extended sensory deficit	Radical nephrectomy	9	141	6.38	3.39	11.69	
Extended sensory deficit	Heminephrectomy	3	42	7.14	2.46	19.01	
Extended sensory deficit	Nephroureterectomy	2	35	5.71	1.58	18.61	

**Table 3:** Logistic regression for flank bulging.

Primary model: age + sex + surgical procedure (reference: radical nephrectomy)													
Variable	OR	95% CI low		95% CI high	*p*		β		SE		Interpretation		
Age (per 10 y)	0.867	0.701		1.073	0.190		−0.141		0.108		No statistically significant association.		
Male sex	0.861	0.524		1.415	0.555		−0.149		0.253		No statistically significant association.		
NSS vs. radical nephrectomy	1.989	1.137		3.480	0.015		0.687		0.285		Higher adjusted odds vs. radical nephrectomy; specific contrast significant.		
Heminephrectomy vs. radical nephrectomy	1.003	0.396		2.538	0.994		0.003		0.473		No statistically significant association.		
Nephroureterectomy vs. radical nephrectomy	1.164	0.429		3.156	0.765		0.152		0.508		No statistically significant association.		
Sensitivity model: additionally adjusted for preoperative HCT and creatinine													
Variable			OR			95% CI low		95% CI high		*p*		β	SE
Age (per 10 y)			0.867			0.701		1.073		0.190		−0.142	0.109
Male sex			0.863			0.522		1.426		0.566		−0.147	0.256
NSS vs. radical nephrectomy			1.972			1.123		3.464		0.018		0.679	0.287
Heminephrectomy vs. radical nephrectomy			0.989352			0.390		2.509		0.982		−0.011	0.475
Nephroureterectomy vs. radical nephrectomy			1.149986			0.418		3.166		0.787		0.140	0.517
Preop HCT (per 5%)			0.986971			0.748		1.303		0.926		−0.013	0.142
Preop creatinine (per 1 mg/dL)			0.89479			0.525		1.524		0.683		−0.111	0.272

**Table 4:** Pairwise comparisons for flank bulging (reference: radical nephrectomy).

Comparison	Unadjusted OR	95% CI Low	95% CI High	Fisher *p*	Holm-Adjusted *p*
NSS vs. Radical nephrectomy	1.896	1.094	3.287	0.024	0.071
Heminephrectomy vs. Radical nephrectomy	1.207	0.496	2.941	0.646	1.000
Nephroureterectomy vs. Radical nephrectomy	1.061	0.396	2.845	1.000	1.000

**Table 5:** Perioperative changes in hematocrit and serum creatinine according to surgical procedure.

Surgical Group	HCT Before, % Median [IQR]	HCT After, % Median [IQR]	*p* Value	Creatinine Before, mg/dL Median [IQR]	Creatinine After, mg/dL Median [IQR]	*p* Value
NSS	40.0 [37.0–43.0]	35.0 [32.0–38.0]	<0.0001	1.00 [0.90–1.10]	1.10 [0.90–1.20]	<0.0001
Radical nephrectomy	38.0 [36.0–41.0]	35.0 [31.0–38.0]	<0.0001	1.10 [0.90–1.30]	1.60 [1.30–1.80]	<0.0001
Heminephrectomy	39.0 [37.0–41.0]	37.0 [35.0–39.75]	<0.0001	1.00 [0.90–1.18]	1.00 [0.90–1.10]	0.588
Nephroureterectomy	35.0 [32.0–39.0]	31.0 [29.5–34.5]	<0.0001	1.00 [0.90–1.25]	1.60 [1.30–1.75]	<0.0001
Entire cohort	39.0 [36.0–42.0]	35.0 [31.0–38.0]	<0.0001	1.00 [0.90–1.20]	1.20 [1.00–1.60]	<0.0001

## Data Availability

The datasets presented in this article are not readily available because protection of personal data in accordance with the European Commission Regulation. Requests to access the datasets should be directed to corresponding author.

## Abbreviations

The following abbreviations are used in this manuscript:

PROM	Patient-reported outcome measure
NSS	Nephron-sparing surgery
CT	Computed tomography
